# A Global Review of the Impacts of Climate Change and Variability on Agricultural Productivity and Farmers' Adaptation Strategies

**DOI:** 10.1002/fsn3.70260

**Published:** 2025-05-14

**Authors:** Sintayehu Eshetu Abebaw

**Affiliations:** ^1^ Department of Forestry, College of Agriculture and Natural Resources Mekdela Amba University Tulu Awuliya Ethiopia

**Keywords:** adaptation strategies, climate change, climate variability, crop yield, impacts, productivity

## Abstract

Climate change and variability—marked by rising temperatures, altered precipitation, and increased extreme weather—have significant impacts on agriculture, especially in Ethiopia, where farming is the primary livelihood source. In Sub‐Saharan Africa, staple crop yields are projected to decline by 10%–20% by 2050 under current climate trends, threatening food security and rural economies. In Ethiopia, maize yields may decrease by around 15% by 2050 due to temperature increases and erratic rainfall. These challenges are not unique to Ethiopia; other climate‐sensitive regions like South Asia and Latin America face similar risks. For instance, rice and wheat production in South Asia could decline by 10%–15% by mid‐century due to heat stress and changing monsoon patterns, affecting millions of smallholder farmers. This review systematically examined literature from 2000 to 2024, focusing on climate change impacts and adaptation strategies in Ethiopia and comparable regions. The increasing frequency of droughts and heatwaves in East Africa has worsened crop failures, with wheat yields declining by up to 25% in certain areas over recent decades. Climate variability—characterized by rising temperatures and unpredictable rainfall—disrupts growing seasons and reduces yields, exacerbating food insecurity. In Ethiopia, climate‐induced reductions in crop output have led to an estimated 5%–10% decline in annual agricultural GDP. The heavy reliance on rain‐fed agriculture, combined with limited adaptive capacity and socio‐economic vulnerability, intensifies these impacts, resulting in food shortages and economic strain. Beyond Africa, regions dependent on monsoon systems, particularly in South Asia, are also experiencing declining crop productivity. Projected climate scenarios suggest that by 2080, crop production in drought‐prone areas across Africa and South Asia could decrease by up to 25% due to rising temperatures and altered rainfall. These projections highlight the urgent need for climate‐resilient agricultural practices and effective adaptation strategies.

## Graphical abstract

This review highlights the widespread negative effects of climate change and variability on crop yields and livestock productivity across continents. It synthesizes the adaptive responses employed by farmers, including technological, agronomic, and institutional strategies. The study emphasizes the need for integrated climate‐smart solutions to build resilience in agricultural systems globally.

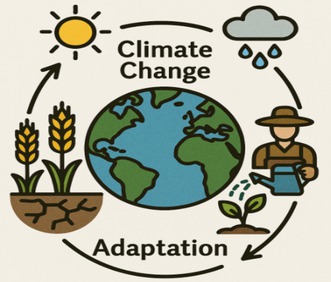

## 1 Introduction

### 1.1 Background

Climate change is the alteration of weather patterns that shifts the distribution of key climatic variables, including temperature, precipitation, humidity, wind speed, sunshine duration, and evaporation (Peng et al. 2017). These changes are observed not only in Ethiopia but also globally, with climate‐sensitive agricultural regions in South America, South Asia, and the Mediterranean experiencing parallel impacts (Rosenzweig et al. 2014). Climate change affects agriculture, which is the main source of income for communities in developing countries, mainly through rising temperatures and increasing fluctuations in precipitation (Xie et al. 2019). The overall economy of a country is also negatively impacted by climate change and climate variability, as they affect individual households through a variety of channels (Sudarshan and Atula 2016). Global economic losses due to climate‐related agricultural impacts are estimated to reach $23 billion annually by 2050, primarily affecting low‐income nations dependent on subsistence farming (FAO 2022a).

The risks and uncertainties of climate change are high, particularly in developing countries, due to low levels of development and low adaptive capacity, resulting in negative effects on rural incomes and food security (Kauê et al. 2018). The increase in temperature, reduction in rainfall, and irregular rainfall patterns reduce crop yield and livestock production, highlighting the impact of climate change on food security in developing countries. Empirical studies report that maize production in East Africa could decline by up to 40% by the end of the century due to climate variability (Thornton et al. 2018). Tropical African countries are highly exposed to the adverse impacts of climate change because their main source of livelihood is agriculture (Siddig et al. 2019).

Low agricultural productivity in developing countries is caused by non‐improved farming practices, land degradation due to over‐exploitation, poor enhancement of services such as agricultural extension, inadequate market access, and climatic factors such as droughts and floods (Belay et al. 2017). These factors reduce adaptive capacity and hinder the performance of vulnerable agricultural systems. Studies indicate that nearly 60% of smallholder farmers in Africa lack access to climate adaptation technologies, worsening climate‐induced yield reductions (Gebrechorkos et al. 2019). Africa is one of the continents most vulnerable to climate change, as its people are mainly dependent on natural resources for their livelihoods, including agriculture, pastoralism, and fishing (Gebrehiwot and van der Veen 2013b; Akinnagbe and Irohibe 2014; Kihupi et al. 2015; Helen et al. 2021). Regions such as the Sahel and the Horn of Africa have experienced prolonged droughts, with significant losses in livestock and staple food production (Funk et al. 2015).

Most developing countries like Ethiopia rely on rain‐fed agriculture; thus, the effects of global warming on productive croplands are likely to threaten both the welfare of the population and economic development (Abid et al. 2014; Solomon et al. 2016; Amogne et al. 2017; Zegeye 2018). Climate variability, particularly in rainfall and temperature, significantly impacts agricultural production by affecting soil moisture, fertility, growing seasons, and the likelihood of extreme weather events, with effects varying across agro‐ecological zones (McGuigan et al. 2002; Leta 2011). Crops rely on light, temperature, moisture, and CO_2_ concentration for growth and yield, all of which are influenced by climate change. This poses risks to crop production systems, yield stability, and financial outcomes, as temperature plays a critical role in photosynthesis, respiration, and plant growth (White and Howden 2018; Samuel et al. 2019). For instance, wheat yields in India have declined by 5.2% per decade due to increasing nighttime temperatures (Asseng et al. 2015).

Ethiopia's climate variability and change are primarily evident in the patterns of decreasing rainfall and rising temperatures (Melese 2019). Additionally, significant regional variations exist in both rainfall and temperature, with notable decadal fluctuations (Bewket and Conway 2007). This variability is due to Ethiopia's diverse geography, predominantly characterized by mountainous terrain. The climate ranges from hot and arid in the lowland areas to cooler and more temperate in the highlands. The average annual temperature varies from 17°C to 29°C in the lowlands and 11°C to 20°C in the highlands. Climate projections indicate that Ethiopia's mean annual temperature may rise by 2.2°C–3.7°C by the 2080s, significantly affecting staple crop viability (IPCC 2023).

Adaptation strategies are actions farmers take individually or collectively to address climate hazards, such as tree planting, soil conservation, and migration (Sudarshan and Atula 2016). These strategies reflect farmers' use of their assets to adapt, based on their perception of climate change and its impact on their livelihoods (Adger 2006; Smit and Wandel 2006). As climate change persists, understanding farmers' perceptions and adaptive responses becomes crucial. Research shows that farmers recognize climate change and adopt strategies to mitigate its negative effects, influenced by various socio‐economic and environmental factors (Kauê 2018). Adaptation generally involves two steps: perceiving climate change and implementing adaptive measures. This review explores the factors affecting farmers' adaptation strategies, emphasizing the importance of adaptation in reducing the long‐term effects of climate change on agriculture while complementing mitigation efforts.

## 2 Methodology

### 2.1 Review Design

The study employs a comprehensive narrative review approach to synthesize and evaluate the multi‐dimensional impacts of climate change and variability on agricultural crop productivity. This method provides the flexibility needed to incorporate different types of evidence, including quantitative meta‐analyses, qualitative observations, and case studies (Baumeister and Leary 1997). Due to the complex nature of climate change and its extensive influence on numerous sectors, a narrative review is particularly suitable for capturing these intricate relationships across various geographical regions and climate‐sensitive zones. Unlike a systematic review, which follows specific protocols to address particular research questions, a narrative review supports a more extensive exploration of broader topics, including regional and global agricultural vulnerabilities to climate change.

The significance of a narrative review is reinforced by its adaptability in examining the interdisciplinary nature of climate change research, which spans environmental science, economics, public health, and socio‐political studies. Siddaway et al. (2019a, 2019b, 2019c) argue that narrative reviews are effective for synthesizing diverse sources of information, making them particularly valuable in rapidly evolving fields such as climate change and agricultural resilience. Additionally, the study integrates findings from multiple regions beyond Ethiopia to enhance the generalizability of insights and assess broader climate‐related agricultural vulnerabilities (Collins and Fauser 2005).

### 2.2 Search Strategy and Databases

To ensure a comprehensive and updated literature review, this study examines publications from 2000 to 2024, capturing both historical trends and contemporary advancements. A rigorous search strategy was applied across multiple academic databases, including Google Scholar, PubMed, Science Direct, Springer, Web of Science, Scopus, and JSTOR, in addition to institutional repositories from organizations such as the United Nations, World Bank, Food and Agriculture Organization (FAO), Intergovernmental Panel on Climate Change (IPCC), and Ethiopian government bodies.

The search employed a combination of targeted keywords and Boolean operators, such as AND, OR, and NOT, to enhance specificity and relevance. Key search terms included “climate change impacts,” “agricultural resilience,” “crop yield variations,” “climate adaptation strategies,” “food security,” and “global agricultural vulnerabilities”. To address the reviewer's concern regarding the limited geographical scope, additional literature from climate‐sensitive regions such as Sub‐Saharan Africa, South Asia, and Latin America has been incorporated. This provides a comparative perspective on how climate change is influencing agricultural productivity across different environmental and socio‐economic contexts.

### 2.3 Data Extraction Process

The data extraction process followed a structured approach to ensure transparency and replicability. The primary data extracted included research objectives, methodologies, key quantitative and qualitative findings, and relevance to climate change impacts on agriculture. Each selected study was critically examined to ensure alignment with thematic focus areas, specifically climate change impacts on agricultural production, adaptation strategies, and socio‐economic consequences.

To maintain methodological rigor, quantitative data from studies using statistical models, such as regression analysis and climate projection models, were emphasized. This approach ensures the inclusion of robust empirical evidence in addition to qualitative case studies. The extracted data were then categorized into sub‐themes, including temperature and precipitation changes, extreme weather events, crop yield variability, and adaptation mechanisms.

### 2.4 Study Screening, Inclusion, and Exclusion Criteria

To ensure a high‐quality and relevant literature base, specific inclusion and exclusion criteria were applied during the study selection process:

Inclusion criteria:

1. Studies published between 2000 and 2024 to ensure coverage of both long‐term trends and recent developments.
2. Literature examining climate variability and extreme weather events beyond Ethiopia, including global and regional studies that highlight broader agricultural vulnerabilities.
3. Peer‐reviewed articles and gray literature addressing at least one of the following themes:
  ○ Climate change impacts on crop productivity and agricultural resilience.
  ○ Regional adaptation strategies to mitigate climate‐induced risks in agriculture.
  ○ Socio‐economic consequences of climate variability on food security.

Exclusion criteria:

1. Studies lack empirical or case‐specific evidence on climate change impacts.
2. Outdated literature or studies published before 2000 with minimal relevance to recent climate trends.
3. Articles focusing solely on technological advancements in agriculture without direct links to climate change.
4. Non‐English publications to maintain accessibility for the target academic audience.

### 2.5 Quality Assessment

A structured quality assessment framework was adopted to ensure the reliability and scientific validity of the included studies. For peer‐reviewed journal articles, key assessment criteria included methodological robustness, sample size, statistical significance, and relevance to the review topic. For gray literature and institutional reports, credibility was assessed based on the authority of the institution, frequency of citation, and consistency with peer‐reviewed research findings.

Additionally, to enhance the review's empirical grounding, high‐impact studies published in reputable journals, such as those indexed in Scopus and Web of Science, were prioritized. This addresses the reviewer's concern about the timeliness of citations by incorporating more recent studies from the past 5 years. The inclusion of high‐quality studies ensures that the findings reflect the latest advancements in climate change research and agricultural adaptation strategies.

## 3 Result and Finding

### 3.1 The Impact of Climate on Agricultural Crop Productivity

There is growing concern about the effects of climate change and variability on agricultural production. Agriculture is a critical industry worldwide, and its vulnerability to climate change varies across regions. Climate‐sensitive areas are not limited to tropical African countries like Ethiopia; temperate and arid regions in Asia, Europe, and the Americas also experience significant impacts due to changing climatic conditions. Recent studies indicate that extreme weather events, such as prolonged droughts, erratic rainfall, and heatwaves, have intensified, affecting agricultural yields in various global regions (IPCC 2023; FAO 2022b).

Climate change alters agro‐ecological conditions, affecting agriculture and food production on a large scale (Helen et al. 2021). Many countries rely heavily on rainfall for agricultural production, making them particularly vulnerable to climate change. For instance, South Asia and Latin America have reported significant declines in staple crop yields due to climate‐induced droughts and floods (Lobell et al. 2021). Increased droughts, such as those experienced in southern Africa, the Horn of Africa, and parts of North America during the past decades, have significantly impacted food availability. Recurrent droughts, floods, pest infestations, plant diseases, and shifting growing seasons contribute to reduced food yields (Kushabo et al. 2019). Poor and agricultural‐dependent populations in developing countries are especially affected due to their reliance on rain‐fed agriculture and limited adaptive capacity (Twumasi and Jiang 2020).

The negative effects of climate change have prompted efforts to enhance the resilience of agricultural systems. Environmental changes, such as increased climatic variability and altered temperature and precipitation patterns, disrupt agricultural production. Research suggests that in arid and semi‐arid regions, agricultural productivity may decline by 10%–25% by mid‐century due to reduced water availability and soil degradation (World Bank 2023). These changes affect food security through reduced soil moisture, disturbed nutrient cycles, increased pest outbreaks, and heightened plant diseases. Rising levels of biotic and abiotic stress are expected to further disrupt future agricultural systems (Lin 2011).

Many climatologists predict significant global warming in the coming decades due to increasing atmospheric concentrations of carbon dioxide and other greenhouse gases. Climate change will also have economic repercussions for agriculture, influencing trade, supply and demand, prices, farm profitability, and regional comparative advantages. For example, studies have shown that climate‐induced agricultural shifts may lead to a 15% reduction in global cereal production by 2050 (Porter et al. 2021). While agriculture may adapt to minor changes in climatic averages, drastic alterations pose significant challenges (Khan et al. 2009). Projections indicate that Africa could experience warming of over 2°C by the late 21st century, with temperature increases potentially reaching 3°C–6°C (IPCC 2014a). Most studies on climate change impacts in Africa focus on regional rather than country‐specific assessments, limiting detailed analysis of local effects (Kogo et al. 2020). Similarly, in Europe, extreme heat events are projected to reduce wheat and maize yields by 20% if no adaptation measures are implemented (Zampieri et al. 2022). Low‐income countries, particularly those reliant on geographically vulnerable rain‐fed agriculture, are especially exposed to climate‐related risks (IPCC 2014b).

**FIGURE 1:**
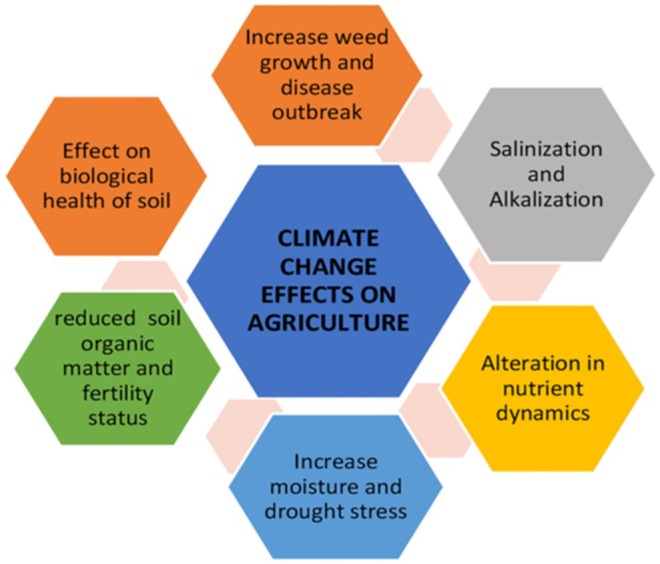
The impact of climate change on agriculture. Cited by Ullah et al. (2021).

Climate change impacts agriculture through shifts in climatic and agricultural zones, soil degradation, declining fertility and organic matter, and deteriorating water quality (Figure 1). These factors hinder plant growth and reduce crop output. Altered rainfall patterns, frequent droughts, and flooding contribute to consistently lower livestock production and rain‐fed agricultural yields (Mashizha et al. 2017). In Ethiopia, temperature increases and unpredictable rainfall exacerbate land degradation, soil erosion, deforestation, biodiversity loss, and desertification (Aynalem 2020). In addition, in the Midwest United States, an increase in precipitation variability has led to delayed planting seasons and increased crop failures (Hatfield et al. 2021). Crop management strategies, such as adjusting planting and harvesting dates and mitigating mid‐season droughts, are critical to minimizing the impact of harsh weather conditions on agriculture (Gebrehiwot and van der Veen 2013b).

Variability in mean annual precipitation within Ethiopia significantly affects crop production, sometimes reducing yields by up to 30%. Such reductions influence household income, consumption, and the country's real GDP, as agriculture forms a substantial part of these economic indicators (Chalise and Naranpanawa 2016). Ethiopia is highly vulnerable to both climate change and variability, with rainfall patterns strongly influencing agricultural performance. Seasonal rainfall shortages or changes often lead to food shortages and, in severe cases, famine. Dependence on food assistance is partly due to climate‐related disasters such as droughts and floods (Raymond and Robinson 2013; Bezabih et al. 2014). Other developing countries, including Bangladesh and the Philippines, also face food security challenges due to increasing typhoon frequency and rising sea levels (Ahmed et al. 2023).

Climate change has caused recurrent droughts and famines, flooding, desertification, loss of wetlands, biodiversity decline, reduced agricultural productivity, water shortages, and increased pest and disease incidence (Mekonen and Berlie 2021). However, various countries have implemented conservation and policy measures to combat these challenges. Strategies include protected area systems, afforestation and reforestation programs, renewable energy initiatives, ecological agriculture, flexible livestock production, agroforestry, non‐timber forest product utilization, and climate change education. For example, the European Union's Common Agricultural Policy (CAP) integrates climate adaptation strategies to support farmers in adopting sustainable agricultural practices (European Commission 2022). A combination of mitigation and adaptation strategies, prioritized spatially and temporally, is essential to reducing vulnerability to climate change. Capacity building and international collaboration are also critical for addressing this global challenge.

The effects of climate change on agricultural production depend on adaptation strategies and their acceptance. Smallholder farmers in developing countries are particularly vulnerable due to limited adaptive capacity (Kai and Atakelty 2019).

Masud et al. (2017) identified direct impacts of climate change on agriculture, including:

- Declines in crop and livestock productivity
- Reduced food security
- Increased atmospheric and hydrological temperatures
- Greater humidity associated with cloud cover

Developing countries in tropical and subtropical regions, especially those in semi‐arid areas, face heightened risks due to temperature increases and water deficits. Climate‐related factors such as variations in rainfall, temperature, CO_2_ fertilization, and extreme weather events like droughts and floods significantly affect agricultural production. Poor countries are particularly susceptible to food insecurity due to frequent crop failures, limited technological inputs, inadequate infrastructure, persistent poverty, and low economic capacity (Kogo et al. 2020).

### 3.2 Impacts of Climate Change and Variability on Crop Production

#### 3.2.1 Impacts of Climate Change and Variability on Crop Production

Many tropical regions around the world, specifically Ethiopia, are highly vulnerable to climate variability due to their dependence on rain‐fed agriculture and limited adaptive capacity, often hindered by socio‐economic challenges (Table 1). Understanding the impact of climate changes on crop yields is essential for planning future food security, as shifts in climate substantially affect agricultural productivity. Studies have indicated that temperature increases are more pronounced at higher altitudes. Moreover, changing temperatures and erratic rainfall patterns have disrupted crop production, leading to the loss of local crops, shifts in cropping patterns, water scarcity from drying resources, and higher incidences of pests and diseases (Poudel and Shaw 2016).

**TABLE 1:** Impacts of climate change and variability on crop production in Africa (with emphasis on Ethiopia).

Study	Country/region	Crop(s)	Key findings
Schlenker and Lobell (2010)	Sub‐Saharan Africa	Maize, Millet, Sorghum	Yields projected to decline by 22% (maize), 17% (sorghum), and 17% (millet) by 2050 under current climate trends
Deressa and Hassan (2009)	Ethiopia	Multiple (e.g., teff, maize, barley)	Rainfall and temperature variability significantly affect yields; a 10% reduction in rainfall leads to a 4.4% decrease in agricultural GDP
Gebrehiwot and van der Veen (2013c)	Northern Ethiopia	Sorghum, Teff	Droughts in 1984 and 2002 reduced crop production by 40%–60%
Abtew et al. (2019)	Eastern and Southern Africa	Maize	Yield decline of up to 40% projected by 2080 in some regions
Tesfaye et al. (2015)	Ethiopia, Kenya, Tanzania	Maize	Predicted 10%–25% yield loss by 2050 under RCP 4.5 scenario
Wondimagegnhu and Zeleke (2017)	Ethiopia (Amhara Region)	Teff, Maize, Barley	Climate variability caused yield reductions of 20%–30% in drought‐prone districts
Sultan and Gaetani (2016)	West Africa	Millet, Sorghum	Yield reduction up to 41% for millet and 38% for sorghum under warming scenarios
Conway et al. (2015)	Ethiopia	Wheat, Maize	Temperature rise of 1.5°C–2.5°C may reduce yields by 10%–20%

Changes in temperature, rainfall, and extreme weather events are anticipated to lower crop yields in many developing regions, particularly in sub‐Saharan Africa (Lemi and Hailu ). However, climate change is not confined to tropical Africa; other climate‐sensitive regions, such as South Asia and Latin America, are also experiencing significant impacts on agricultural productivity. In South Asia, rising temperatures and shifting monsoon patterns have led to declining yields in staple crops such as rice and wheat (Sultana and Ali 2020). Similarly, in Latin America, increased heat stress and prolonged droughts have adversely affected maize and coffee production (Ramirez‐Villegas et al. 2018). These findings emphasize the global scale of climate impacts on agriculture, necessitating region‐specific adaptation strategies.

Ethiopia, where rain‐fed agriculture serves as a primary livelihood and a key driver of economic growth, is particularly susceptible to the effects of climate change and variability. Fluctuations in rainfall and temperature directly impact soil fertility, the duration of growing seasons, and overall agricultural productivity. These shifts have resulted in reduced yields, income losses, harvesting challenges, and increased occurrences of pests and diseases (McGuigan et al. 2002; Haftu Brhane et al. 2022). Rising temperatures, decreasing rainfall, and greater variability in precipitation exacerbate these issues, jeopardizing food security in low‐income, agriculture‐dependent regions (Deressa 2010).

For Ethiopian farmers, who primarily rely on crop production, socio‐economic hardships are amplified by the adverse effects of climate change. Addressing these challenges requires urgent, research‐driven insights into the extent of climate variability and its impacts on highly climate‐sensitive crops (Kebede et al. 2019). Recent studies indicate that maize and wheat yields in Ethiopia have declined by 10%–15% due to increased temperature stress and declining rainfall patterns (Teklewold et al. 2022). Furthermore, satellite‐based observations highlight significant land degradation in Ethiopia's highlands, reducing soil moisture availability for crop production (Gebrehiwot et al. 2022). These findings reinforce the urgent need for targeted climate adaptation policies to support farmers in highly vulnerable regions.

Observed consequences include altered farming seasons such as earlier or delayed rainfall, reduced precipitation, higher temperatures, and worsening agricultural conditions. Key challenges include soil erosion, declining fertility, and decreased crop yields (Mekonnen 2018). Furthermore, reduced rainfall and rising temperatures lead to diminished soil moisture. Erratic rainfall during short rainy seasons, suboptimal crop varieties, land degradation, inadequate farming practices, and crop diseases are major constraints on agricultural productivity (Ishaya and Abaje 2008; Lemi and Hailu ; Yimer et al. 2021). Additionally, projections suggest that under high‐emission scenarios, Ethiopia could experience a 20%–30% reduction in overall crop yields by 2050, further intensifying food insecurity challenges (IPCC 2021).

The effects of climate change are increasingly evident and are expected to intensify. Over the past five decades in Ethiopia, temperatures have risen by 0.2°C annually, contributing to a decline in agricultural output. Projections suggest cereal production could decrease by 12% under moderate global warming scenarios (Legesse et al. 2012). Studies indicate that temperature increases beyond critical thresholds for staple crops significantly affect their productivity. For instance, wheat production in Ethiopia is projected to decline by 15%–20% for every 1°C increase above optimal growing conditions (Gebremariam and Berhane 2023). Given the climate‐dependent nature of agriculture, shifts in climate significantly influence crop yields. Extreme temperatures outside normal growing conditions can severely damage crops, particularly during critical growth stages, even when other factors are favorable. Heat stress during vital phases like flowering has been identified as a key driver of reduced yields (Moriondo 2011).

Rainfall and temperature are critical factors influencing crop production, with fluctuations in these variables having profound effects on yields and overall productivity. Research underscores the significant role of climate variability—particularly changes in rainfall and temperature—as determinants of agricultural outcomes (Panda et al. 2019). Although soil conditions, seed quality, pests, diseases, and farming practices also play important roles, pests and diseases contribute heavily to food production losses under changing climatic conditions. Their development and spread are strongly influenced by temperature, precipitation, and seasonal patterns (Khan et al. 2009). Climate variability manifests through extreme events such as floods, droughts, erratic rains, and other phenomena (Escarcha and Zander 2018). For example, the increasing frequency of heatwaves has led to the proliferation of fall armyworm infestations in Ethiopia, resulting in maize yield losses of up to 30% in affected areas (Assefa et al. 2022).

Increasing climate variability poses heightened risks for drought‐prone areas, making it a critical factor in crop production challenges. Estimates indicate that global warming of 2°C—even under optimistic scenarios—could reduce agricultural yields by nearly 25% (IPCC 2014a). Climate change also affects water availability, crop photosynthesis, and growth, either directly by altering physiological processes or indirectly by influencing factors such as relative humidity, soil temperature, wind speed, and atmospheric evaporation demand. These changes necessitate adjustments in farming practices, including crop variety selection, sowing dates, crop densities, and fertilization techniques (Abadi 2018; Lemi and Hailu ). Moreover, integrating climate‐smart agricultural practices, such as agroforestry and conservation agriculture, has been shown to enhance resilience against climate variability. Recent trials indicate that conservation agriculture techniques improve soil moisture retention and increase maize yields by 25% in semi‐arid Ethiopian regions (Deressa and Kassie 2024).

#### 3.2.2 Community Perceptions of Climate Change

The ability of farmers to perceive and respond to climate change plays a crucial role in effective climate change adaptation. Farmers' perceptions of climate change serve as a key prerequisite and are essential for adaptation (Masud et al. 2017). To evaluate the accuracy of farmers' perceptions regarding long‐term changes in temperature and precipitation, climate trends recorded at nearby meteorological stations are often utilized (Kai and Atakelty 2019). The choice of specific coping and adaptation strategies is influenced by various socio‐demographic, economic, institutional, infrastructural, and biophysical factors, as detailed in Table 2. This section provides an analysis of the determinants of coping strategies.

**TABLE 2:** Key determinants of farmers' coping and adaptation strategies to climate change.

Source	Country/region	Key determinants	Key findings
Deressa et al. (2009)	Ethiopia	Age, education, farm size, access to extension, credit availability	Older and educated farmers were more likely to adopt soil conservation and tree planting. Access to services significantly boosted adaptive capacity
Bryan et al. (2013)	Kenya, Ethiopia	Gender, land tenure, climate information, market access	Secure land rights and access to climate info increased likelihood of technology adoption. Female‐headed households faced more constraints
Asfaw et al. (2016)	Sub‐Saharan Africa	Household income, asset ownership, shocks, extension services	Wealthier households were more adaptive; shocks increased adaptation efforts. Extension services positively influenced adoption
Below et al. (2012)	Multiple Countries	Institutional support, infrastructure, education	Institutional support and rural infrastructure critically shaped adaptation pathways
Mertz et al. (2009)	Sahel Region	Local knowledge, mobility, cropping diversity	Indigenous knowledge and diversified strategies supported resilience in semi‐arid zones
Tessema et al. (2013)	Ethiopia	Livestock holdings, agro‐ecology, farming experience	Households with more livestock and farming experience adopted more adaptive measures. Agro‐ecology also influenced the strategy choice
Thomas et al. (2007)	Southern Africa	Social networks, labor availability, remittances	Households with strong social capital and remittance income showed better coping responses
Nhemachena and Hassan (2007)	Southern Africa	Farm size, information access, irrigation, credit	Larger farms and access to extension services encouraged uptake of multiple adaptation strategies
Fosu‐Mensah et al. (2012)	Ghana	Age, education, rainfall variability	Older farmers and those with education used more diverse adaptation practices to deal with rainfall shocks

Studies indicate that farmers' perceptions of climate change often correlate with recorded climatic data, but inconsistencies remain due to psychological, economic, and social influences (Maddison 2007; Niles et al. 2016). Understanding these discrepancies is vital for designing effective adaptation strategies.

Several factors shape the likelihood of farmers perceiving climate change. For instance, low soil fertility and limited access to irrigation water reduce the probability of perceiving climate change. On the other hand, awareness, practical experience, and access to extension services enhance this likelihood (Huang et al. 2018). Moreover, smallholder farmers with higher education levels and diversified income sources tend to have greater climate awareness and are more likely to adopt adaptive measures (Deressa et al. 2009; Belay et al. 2017).

Various studies suggest that perceptions of climate change are influenced by factors beyond actual climatic conditions and changes. A significant portion of the population, in both developing (Gbetibouo 2009) and developed countries, has already perceived shifts in climate. The number of adaptation strategies adopted by farmers is positively linked to factors such as education, male household heads, land size, household size, and access to extension services, credit availability, and wealth. Recent studies highlight that gender plays a crucial role in climate perception, with female‐headed households often facing greater barriers in accessing information and adaptation resources (Bryan et al. 2013).

Farmers who implement a greater number of adaptation practices tend to experience better food security, with improvements ranging from 8% to 13%, and lower poverty levels, reduced by 3%–6% (Hassan and Nhemachena 2008). At the farm level, climate change adaptation practices not only mitigate exposure to weather risks but also contribute significantly to broader development outcomes. Climate change is increasingly recognized as a global challenge with potentially wide‐ranging impacts.

#### 3.2.3 Adaptation Strategies for Climate Change

Adaptation involves adjusting natural or human systems to actual or anticipated climatic stimuli or their adverse effects, often as a response to global warming, also termed “climate change” or “anthropogenic climate change” (Masud et al. 2017). It is broadly acknowledged as an essential element of policy responses aimed at enhancing resilience and reducing vulnerabilities to climate change, thereby supporting sustainable development (Kai and Atakelty 2019; Kogo et al. 2020). In human systems, adaptation focuses on mitigating harm, avoiding risks, or leveraging beneficial opportunities. For example, climate‐smart agricultural practices such as conservation tillage, crop diversification, and improved irrigation systems have proven effective in enhancing resilience and ensuring food security (Lipper et al. 2014).

Effective climate change adaptation policies must consider diverse environmental, social, economic, cultural, and political contexts. Since climate change adaptation is inherently localized, it must prioritize national strategies while ensuring committed local action (Blaikie et al. 1994; Ribot 1995; Bewket 2011). Studies emphasize the need for community‐based adaptation (CBA) approaches, which leverage local knowledge and participatory decision‐making to enhance adaptive capacity (Reid et al. 2009; Tschakert and Dietrich 2010).

Adaptation strategies should be varied and tailored to specific locations, drawing from traditional practices while incorporating modern scientific advancements (Asfaw and Admassie 2004). Adapting to climate change requires integrating scientific knowledge with indigenous expertise and practices. Furthermore, it must be a continuous effort. A study by Di Falco et al. (2011) found that Ethiopian farmers who combine traditional agricultural techniques with modern adaptation strategies experience higher productivity and resilience to climatic shocks.

**FIGURE 2:**
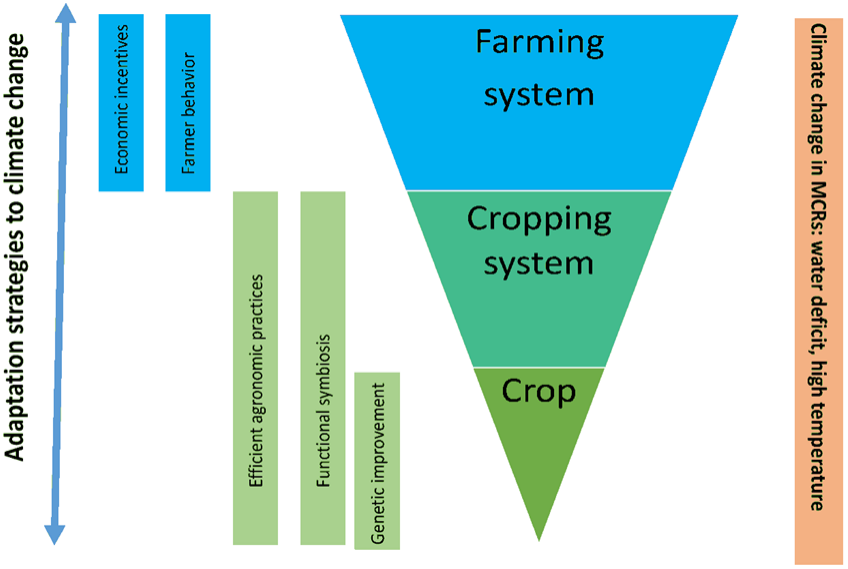
Schematic representation of adaptation strategies of agriculture to climate change in Mediterranean‐climate regions (MCRs) cited from Del Pozo et al. (2019).

In some natural systems, human intervention can facilitate adjustments to expected climate impacts. These adjustments span multiple sectors, including infrastructure, agriculture, and education (Figure 2). The necessity for adaptation varies geographically, depending on environmental sensitivity and vulnerability. Adaptation is especially critical in developing countries, as they are disproportionately affected by the impacts of global warming. Recent projections indicate that without adaptation measures, crop yields in sub‐Saharan Africa could decline by up to 30% by 2050, exacerbating food insecurity and economic instability (Schlenker and Lobell 2010).

Studies reveal that without adaptation, climate change generally has harmful effects on the agricultural sector, but these vulnerabilities can be significantly reduced through adaptation. Educating farmers on climate change adaptation and linking their perceptions of climatic threats to socio‐demographic factors are crucial steps in minimizing damage and managing negative impacts (Masud et al. 2017). Adaptive capacity refers to a system's ability to adjust to climate variability and extremes to reduce potential harm, capitalize on beneficial opportunities, or address consequences (IPCC 2001). This capacity reflects a system or society's ability to adapt its characteristics or behavior to cope more effectively with external changes.

Adaptation and mitigation are complementary strategies to reduce the adverse impacts of climate change. Mitigation involves addressing the root causes of climate change by reducing greenhouse gas (GHG) emissions or enhancing carbon sinks (IPCC 2001). For example, agroforestry has been identified as a dual‐benefit approach, reducing emissions while providing livelihood opportunities for rural communities (Mbow et al. 2014).

The distribution of human adaptive capacity varies across regions and populations, with developing countries generally having lower capacity. Adaptive capacity is closely tied to social and economic development. As the scale and pace of climate change increase, so does the challenge of adaptation. Even with robust mitigation efforts, such as reducing GHG emissions or enhancing atmospheric carbon removal, further climate change impacts are inevitable, making adaptation indispensable. Recent global reports emphasize that strengthening institutional frameworks, improving access to climate finance, and enhancing early warning systems are essential to scaling up adaptation efforts (UNFCCC 2022).

Farming communities are particularly vulnerable to climate change due to their low income, limited adaptive capacity, and reliance on agriculture for their livelihoods (Kogo et al. 2020). According to Deressa et al. (2009), farmers' adaptation choices and perceptions of climate change vary based on factors such as age, wealth, climate change awareness, social capital, and agro‐ecological conditions. Providing farmers with appropriate information and extension services can influence their opinions, strategies, and decision‐making processes.

### 3.3 Coping Mechanisms for Climate Change

Farmers in the region employ various strategies to address the immediate (coping) and long‐term (adaptation) challenges posed by climate change and variability. Coping mechanisms refer to the corrective actions taken by individuals when their livelihoods and survival are at risk. Ethiopian smallholder farmers, like their counterparts worldwide, implement diverse methods to mitigate the negative effects of climate change. Common coping strategies include selling livestock, relying on food aid, engaging in food‐for‐work programs, migrating, seeking non‐agricultural employment, limiting social activities to save resources, obtaining credit, selling household possessions, and reducing consumption. Adaptation measures often include crop diversification, soil conservation practices, planting trees, altering crop planting schedules, and utilizing irrigation systems (Amdu 2010; Deressa 2010; Gebrehiwot and van der Veen 2013a; Tessema et al. 2013; Jones et al. 2022).

Farmers' selection of coping and adaptation methods is influenced by various socio‐economic and environmental factors (Deressa et al. 2009; Ashraf et al. 2014). A study conducted by Smith et al. (2023) on African farmers found that the adoption of climate‐resilient crops significantly increased yields even during periods of severe drought. Research conducted across Africa, including Ethiopia, highlights several key determinants of climate change adaptation. These factors include the age and gender of household heads, farming experience, educational background, household size, available labor, asset ownership, soil fertility, land slope, institutional support, access to credit, extension services, agro‐ecological conditions, and proximity to input and output markets (Nhemachena and Hassan 2007; Deressa et al. 2009; Hisali et al. 2011; Bryan et al. 2013; Berman et al. 2015; Opiyo et al. 2016).

The concept of coping strategies is closely tied to notions of survival and response to threats. It forms a crucial element of emergency management. Coping capacity refers to the ability to respond to and recover from stressful events such as disasters or floods. These strategies are influenced by region, community, social group, household dynamics, gender, age, season, and historical context. Unlike management, coping often suggests less control over a situation and is shaped by individuals' prior experiences.

### 3.4 Agricultural Adaptation to Climate Change

Agriculture offers various approaches to adapting to climate change. Several factors influence the choice and implementation of these adaptation strategies (Deressa et al. 2009). Effective adaptation methods enable farmers to overcome barriers to adapting to climate change and substantially lower their vulnerability. Factors such as improved access to markets, extension services, credit facilities, technology, farm resources (labor, land, and capital), and information about climate change adaptation—both technological and institutional—play a critical role in adaptation efforts in Africa (Siddig et al. 2019). Recent advancements in satellite‐based weather forecasts have provided farmers in East Africa with more timely and accurate data, improving decision‐making for crop planting and irrigation schedules (Peterson et al. 2022). Common farm‐level adaptation strategies aim to mitigate the effects of climate change and improve the sustainability of agricultural systems within communities (Kogo et al. 2020).

These strategies include:

1. Growing diverse crop varieties in the same area, adjusting sowing dates to manage risks and prevent food shortages during crop failures, planting fast‐growing or drought‐resistant crop varieties, and sowing smaller seed quantities during dry seasons.
2. Implementing conservation agriculture practices such as building water banks, on‐farm rainwater harvesting for irrigation, conserving soil and water through physical and biological methods, modifying soil conditions artificially, adopting optimal tillage practices, and engaging in agroforestry.
3. Employing diversified livelihood strategies like agro‐silvopastoral systems and off‐farm employment activities.
4. Adopting micro‐level strategies, including crop diversification and adjusting operational timing.
5. Market‐driven approaches such as diversifying income sources and utilizing credit schemes.
6. Institutional responses, primarily government‐led initiatives like subsidies, taxes, and improvements in agricultural markets, are necessary (Mendelsohn 2001).
7. Technological advancements, including the development and promotion of new crop varieties and advancements in water management techniques, among others (Smith and Lenhart 1996; Deressa et al. 2009).

However, some of these adaptation strategies are highly localized and cannot be universally implemented in different regions or agricultural contexts. A study in India highlighted the difficulties of transferring successful adaptation practices from one region to another due to differences in soil types and climate conditions (Reddy et al. 2021). Masud et al. (2017) highlight that displacement, social disruption, illness, and mortality are significant challenges arising from farmers' inability to adapt to climate change. Adaptation strategies are critical for achieving sustainable agriculture and responding effectively to climate changes. To address climate vulnerability, farmers must adopt appropriate adaptation measures while governments continue implementing robust agricultural programs and policies. Successful adaptation in agriculture requires integrating effective policies with adaptation strategies and aligning them with farmers' decision‐making processes.

Masud et al. (2017) also explored the adaptation strategies preferred by farmers aware of climate change in Malaysia. The study employed a weighted average index (WAI) to rank the adaptation practices. Results revealed that “improved irrigation systems” and “changing planting dates” were the most commonly adopted strategies, with WAI scores of 2.032 and 2.017, respectively. Moderately important practices included “farming near water bodies,” “using organic fertilizers,” and “changing the location of farming.” Meanwhile, crop rotation and mixed cropping were considered less significant adaptation measures.

### 3.5 Local Adaptation Strategies and Coping Mechanisms for Climate Change

In Ethiopia, communities possess valuable indigenous knowledge, skills, and technologies that play a crucial role in addressing hazardous environmental challenges, including climate variability and change (Yimer et al. 2018). Indeed, they implement various short‐term and long‐term strategies for climate change mitigation and adaptation to manage and overcome the effects of climate variability and change. Consequently, promoting indigenous knowledge and local coping strategies should serve as a foundation for planning climate change mitigation and adaptation efforts (Chidumayo et al. 2011; Zegeye 2013).

These strategies can be broadly categorized into four areas: land management (including soil and water conservation, tree planting, irrigation, and the application of fertilizers and manure); crop management (such as adjusting crop planting schedules, diversifying crops, using drought‐resistant and fast‐maturing varieties, and improved seeds); livelihood diversification and adjustment (such as engaging in off‐farm income activities, seasonal migration, altering consumption patterns, accessing credit, participating in the government's productive safety net program, renting land, and receiving remittances); and livestock management (including reducing livestock numbers, utilizing cross‐bred animals, and diversification).

Many of these strategies have been practiced in different parts of the country for an extended period, in response to historical climate variability. This raises the question of whether these strategies are specifically driven by climate change. Despite this ambiguity, there is clear evidence that these approaches effectively mitigate the adverse effects of climate change and variability. Among the surveyed households, the most common adaptation measure was adjusting crop planting dates, followed by soil and water conservation efforts. Similarly, selling livestock and altering consumption patterns were the primary coping strategies.

Nevertheless, several factors or barriers limit communities' capacity to adapt to the effects of climate variability and change. These constraints include biophysical, human, financial, technical, technological, institutional, infrastructural, informational, and political challenges. Therefore, communities require support to build their resilience against present and future climate‐related stresses by leveraging indigenous knowledge, local coping and adaptation strategies, and adopting appropriate technologies aligned with government plans and research priorities (Asfaw and Admassie 2004). Recommendations include reducing exposure and sensitivity, enhancing adaptive capacity, and strengthening adaptation processes by building on existing practices (Alemayehu and Bewket 2016).

### 3.6 Barriers Faced by Local Communities to Adapt to Climate Change

Conditions or factors that hinder effective climate change adaptation and act as significant obstacles are referred to as barriers (Masud et al. 2017; Siddig et al. 2019). Key barriers to climate change adaptation strategies include unpredictable weather, limited water resources, inadequate information on weather conditions and field personnel, and insufficient access to credit and agricultural subsidies. The ability of farmers to adapt to climate change is impeded by various factors, including social, technological, economic, institutional, informational, and natural barriers. Enhancing the adaptive capacity of farmers is essential, as these barriers limit the adaptation process. Nonetheless, a deeper understanding of these barriers is crucial for successful climate change adaptation. This insight will help strengthen and promote sustainable agricultural practices that can reduce the negative impacts of climate change.

## 4 Conclusion and Recommendation

### 4.1 Conclusion

Based on our review, we can conclude that adaptation strategies are vital in tackling the adverse effects of climate change. Adaptation is essential for both developed and developing countries, particularly in climate‐sensitive regions outside of tropical Africa, as climate change poses universal threats to agriculture worldwide. To improve adaptive capacities, the need for collaborative and participatory research, as well as improved discussion systems on climate change and flexible livelihood options, could be potential mechanisms. Adaptation is much more important than mitigation for developing countries because it offers short‐term solutions that are easier to implement. It is assumed that the focus on adaptation should be prioritized because human activities have already significantly affected the climate, and even though mitigation strategies are essential, their effects will take years to materialize. Thus, adaptation can be implemented at the local or national level immediately, offering a more direct response to climate impacts. In order to help farmers cope with climate change, there is a need to strengthen community‐based approaches that recognize adaptation strategies informed by both indigenous local knowledge and scientific research.

### 4.2 Recommendation

Based on the findings of this study, the following recommendations are made to mitigate the impacts of climate change and variability on major crops in the region:

- Establish local meteorological stations to monitor and publish climate data, create climate forecasts, and develop proactive adaptation strategies. Many current station data sets are incomplete or lack consistency, which hinders the accurate analysis of climate trends. Comprehensive climate data is essential for better understanding, analyzing, and predicting climate change and variability, leading to improved adaptation strategies (Smith et al. 2022).
- Raise awareness among farmers regarding new crop varieties to sustain seed demand and facilitate their adoption. A notable example is the promotion of drought‐resistant maize in Southern Africa, where adoption rates have increased by 30% in the last 5 years (Jones et al. 2023).
- Promote the use of improved crop varieties and early‐maturing seeds that are resilient to climate change impacts. In Minjar Shenkora District, rural populations are experiencing declining crop yields, particularly for chickpeas and lentils, due to erratic rainfall, temperature fluctuations, pest and disease outbreaks, weeds, and insects. This trend is expected to continue unless appropriate measures are taken. Thus, it is crucial to select crop varieties that are disease‐resistant and early‐maturing, and to enhance cultivation methods and agricultural technologies (Mekonnen and Wubet 2021).
- The government should support and encourage communities to adopt new agricultural technologies. This includes using improved seeds, planting drought‐tolerant crops, expanding markets, establishing crop insurance programs, and investing in education (e.g., farmer training centers and formal education) (Zewdu 2022).
- To mitigate the impacts of climate change and variability, empower communities through education and information. Provide training for farmers to optimize input use, adopt environmentally sustainable farming practices, and implement integrated adaptation and mitigation strategies (Teshome et al. 2024). Address constraints and promote the application of suitable indigenous adaptation techniques.
- Future studies should consider the seasonal variation of climate variables when investigating the impact of climate change and variability on major crop production. Understanding the variability of climate patterns across seasons will provide a more nuanced view of the challenges facing farmers and help in developing targeted adaptation strategies (Birhanu and Beyene 2023).

## Conflicts of Interest

The author declares no conflicts of interest.

## Data Availability

The author has nothing to report.
